# Pod Mod Electronic Cigarettes—An Emerging Threat to Public Health

**DOI:** 10.1001/jamanetworkopen.2018.3518

**Published:** 2018-10-05

**Authors:** Tory R. Spindle, Thomas Eissenberg

**Affiliations:** Behavioral Pharmacology Research Unit, Johns Hopkins University School of Medicine, Baltimore, Maryland; Center for the Study of Tobacco Products, Department of Psychology, Virginia Commonwealth University, Richmond

There has been a recent and substantial shift in the tobacco and/or nicotine product landscape. Tobacco cigarettes, after dominating the market in the last century, have been surpassed by electronic cigarettes (e-cigarettes) as the most commonly used tobacco product among youths.^1^ E-cigarettes are a class of products that use a heating element to aerosolize a liquid for users to inhale; the liquid typically contains nicotine, various flavorants, and solvents such as propylene glycol and/or vegetable glycerin. A study by McKelvey and colleagues^2^ highlights the ascension of a new iteration of e-cigarettes known as pod mods and corroborates numerous reports in popular media that have documented the increase in popularity of one pod mod in particular, JUUL, among adolescents and young adults.

The study assessed the use of pod-based e-cigarettes, conventional or non-pod mod e-cigarettes, and tobacco cigarettes among California high school students (N = 445). As in larger surveys, ever product use was higher for conventional e-cigarettes relative to tobacco cigarettes, but participants reported using pod-based e-cigarettes in the past 30 days and 7 days at the highest frequency. Consistent with another recent study of adolescents,^3^ approximately 60% of those who reported ever use of pod-based e-cigarettes also reported past 30-day use (37% also reported pod-based e-cigarette use in the past 7 days). Also, similar to national youth surveys,^4^ a much smaller proportion of ever users of conventional e-cigarettes and tobacco cigarettes also reported past 30-day (<30%) or 7-day use of those products (<20%). Thus, crucially, youths who try pod-based e-cigarettes appear to be far more likely to become regular users compared with those who try other e-cigarettes or tobacco cigarettes, although longitudinal studies are necessary to confirm this assertion.

Several features of pod-based e-cigarettes have likely contributed to their increase in popularity among youths. First, these devices can be used discretely, as they easily are concealable and produce relatively little visible aerosol (or vapor). These qualities make them ideal to use in locations where e-cigarette use is forbidden such as on school grounds as is often reported in popular media articles. Second, as with conventional e-cigarettes, they come in a variety of flavors such as mango, crème brûlée, cool mint, and fruit medley. Third, innovative marketing campaigns on social media platforms^5^ undoubtedly helped pod-based e-cigarettes to capture the majority (72%) of the US e-cigarette market.^6^ Third, pod-based e-cigarettes contain high levels of nicotine, the primary addictive constituent of tobacco products. Product labeling claims pods contain 59 mg/mL of nicotine, yet actual concentrations can be much higher (eg, 75 mg/mL).^7^ The nicotine contained in pods is predominantly in a protonated form (referred to as nicotine salts), unlike conventional e-cigarette liquids that contain a nonprotonated or free base form of nicotine. Under some conditions, pod-based e-cigarettes can deliver nicotine to pod mod–naive cigarette smokers at levels that are comparable with their own brand of cigarettes^8^ but nicotine delivery also appears to vary widely among these individuals.^7,8^ More research is needed to elucidate the nicotine delivery profile of experienced pod-based e-cigarette users and the comparative pharmacokinetics of protonated and nonprotonated nicotine from pod mods and other e-cigarettes.

Whatever the cause, the seemingly widespread use of pod mod e-cigarettes among youths could have detrimental consequences for public health. Although e-cigarettes typically emit less toxicants than tobacco cigarettes, they are not toxicant-free. When heated and aerosolized, the contents of e-cigarette liquids can undergo thermal decomposition, resulting in the formation of toxic and/or carcinogenic carbonyls, furans, and other harmful compounds.^9^ E-cigarette aerosols can also contain other toxicants such as reactive oxygen species (free radicals) and heavy metals.^9^ Potential adverse health effects of e-cigarette use can include respiratory and mucosal irritation and/or inflammation, acute adverse cardiovascular effects, and increased oxidative stress.^9^ Repeated nicotine exposure can also influence the development of the adolescent brain, particularly in the prefrontal cortex, which does not reach maturity until the mid 20s.^9^ Finally, nicotine-naive adolescents who try e-cigarettes are more likely to initiate tobacco smoking.^9^ In the study by McKelvey and colleagues,^2^ among ever users of any tobacco product, 25% reported ever use of pod-based e-cigarettes, conventional e-cigarettes, and tobacco cigarettes, while only 2% reported ever use of pod-based e-cigarettes only. Thus, pod-based e-cigarettes may be used at a high rate in combination with other addictive and potentially more harmful products. To determine to what extent these devices promote the use of other tobacco products, national longitudinal surveys must quickly begin to include items assessing pod mod use.

Some of the same qualities of pod-based e-cigarettes that make them dangerous for youths suggest promise as an effective smoking cessation aid. For example, pod mods can deliver high levels of nicotine capable of suppressing adverse symptoms associated with nicotine and/or tobacco abstinence and are relatively easy to use. Moreover, design features of pod mods may increase the overall safety profile relative to cigarettes and some other e-cigarettes. For instance, JUUL restricts users to 5-second puffs,^7^ the heating element is disabled at 215°C,^7^ and users cannot modify the power output. Further, unlike most e-cigarettes, users do not add e-cigarette liquid to the JUUL but rather each pod comes prefilled. Overall, standardization of e-cigarette device and liquid features and allowable puffing behaviors could help mitigate toxicant emissions and deter unintended uses for these products, but more research is needed to establish the ideal parameters for minimizing harm. These safety features of pod-based e-cigarettes notwithstanding, these devices should not be in the hands of adolescents, as no amount of long-term nicotine exposure is likely safe for youths.

If pod mods or e-cigarettes are to have any benefit to public health, youths’ use and access to these products must be reduced drastically. The US Food and Drug Administration recently acted to deter the sale of pod-based e-cigarettes to underage youths both online and in retail locations. While this is an important first step, efforts to restrict marketing that targets youths, particularly on social media, and limit the availability of attractive flavors could also be effective in curtailing pod-based e-cigarette use. Given that most smokers who switch to e-cigarettes begin with either tobacco or menthol flavors,^10^ other flavor categories that attract youths are likely unnecessary. Tellingly, only 1 individual in the study by McKelvey and colleagues^2^ reported that their first pod was tobacco flavored. JUUL recently began selling lower nicotine concentrations for 2 flavors (tobacco and cool mint), but not for their other fruit and dessert flavors that may directly appeal to adolescents. If left unaddressed, the combination of appealing flavors and high nicotine concentrations in pod mods could undermine tobacco control efforts.

Importantly, the numerous other pod mods on the market should not be overlooked. These other devices, including Bo, Kwit Stick, Mistic, Rubi, and Suorin share similar design features to JUUL and their liquids may also contain pronated nicotine. Indeed, protonated nicotine used to refill some pod mods can be purchased in concentrations as high as 60 mg/mL, with flavors like cotton candy, donut cream, and gummy bear. Additionally, pod mods containing Δ9-tetrahydrocannabinol (THC), the primary psychoactive constituent of cannabis, have emerged. Youths’ access and use of these and other pod mods should be monitored and scrutinized. Without swift public health and regulatory action, pod mods and e-cigarettes could contribute to a new epidemic of nicotine addiction that may well be accompanied by novel health threats.
